# Understanding fibroblast-immune cell interactions *via* co-culture models and their role in asthma pathogenesis

**DOI:** 10.3389/fimmu.2023.1128023

**Published:** 2023-02-23

**Authors:** F. Thiam, S. Al Yazeedi, K. Feng, S. Phogat, E. Demirsoy, J. Brussow, F. A. Abokor, E. T. Osei

**Affiliations:** ^1^ Department of Biology, University of British Columbia, Kelowna, BC, Canada; ^2^ Centre for Heart Lung Innovation, St. Paul’s Hospital, Vancouver, BC, Canada

**Keywords:** lung fibroblast, lung immune cells, *in vitro* co-culture models, airway inflammation, airway fibrosis, asthma

## Abstract

Asthma is a chronic lung disease involving airway inflammation and fibrosis. Fibroblasts are the main effector cells important for lung tissue production which becomes abnormal in asthmatics and is one of the main contributors to airway fibrosis. Although fibroblasts were traditionally viewed solely as structural cells, they have been discovered to be highly active, and involved in lung inflammatory and fibrotic processes in asthma. In line with this, using 2D and 3D *in vitro* co-culture models, a complex interaction between lung fibroblasts and various immune cells important for the pathogenesis of asthma have been recently uncovered. Hence, in this review, we provide the first-ever summary of various studies that used 2D and 3D *in vitro* co-culture models to assess the nature of aberrant immune cell-fibroblast interactions and their contributions to chronic inflammation and fibrotic mechanisms in asthma pathogenesis.

## Introduction

Asthma is a chronic inflammatory lung disease that is a major global health burden with an estimated prevalence of 357.4 million (as of 2019) (1). Asthma is caused by allergic (e.g., pollen, house dust mite etc.), and non-allergic (e.g., exercise, cold air, microbial exposure, pollution etc.), triggers and is a highly heterogenous lung disease which involves chronic inflammation, fibrosis, and remodeling in the large airways that leads to variable airflow limitation (2, 3). There is currently no cure for asthma and most pharmacologic treatments of the disease can mainly manage the inflammatory symptoms while the fibrotic lesions in the airways are largely irreversible (4, 5). Therefore, there has been an increased interest in understanding the underlying causes of fibrotic mechanisms in asthma in a bid to find new therapeutic targets.

The main effector cell involved in airway-fibrosis is the fibroblast which is mainly responsible for extracellular matrix (ECM) production in the lungs (6–9). In the pathobiology of asthma, fibroblasts respond to the release of various mediators (e.g., cytokines) by the damaged airway epithelium upon allergen exposure. These epithelial-cytokines also recruit different immune cells such as eosinophils, mast cells and T_H_2 cells which then communicate with fibroblasts to add to the induction of various asthma features (10–12). In addition, there is epithelial-release of fibrogenic mediators (e.g., transforming growth factor (TGF-β)) leading to fibroblast activation and differentiation to myofibroblasts (13–16). Myofibroblasts are highly synthetic cells that vigorously produce and increase the deposition of ECM proteins (e.g., collagen), responsible for asthmatic airway fibrosis (17–19).

Lung fibroblasts were traditionally thought of as structural cells primarily responsible for ECM synthesis and organization (20–22). However, it is now clear that fibroblasts play significant roles in the immune system as they are now known to respond to and produce a variety of inflammatory signals in addition to growth factors which can influence immune cell behaviour (22). This critically points to the ability of fibroblasts to potentially communicate with immune cells in various mechanistic aspects of different lung diseases such as asthma.

To assess how immune cells and fibroblasts interact, researchers have utilized a plethora of complex 3-dimensional (3D) *in vitro* models. These *in vitro* models allow scientists to recreate and mimic the complex 3D spatial orientation of cells in the *in vivo* environment and examine the aberrant cell interactions that occur in chronic lung diseases such as asthma. The various models used range from simple co-culture experiments to more complex (3D) tricultures and microfluidic lung-on-a-chip systems. Co-culturing fibroblasts with various immune cells in these systems, allows the investigation of specific mechanisms involved in lung fibroblast-immune cell crosstalk. This in turn allows for the advancement of studies into targeted treatments and medications to reduce disease symptoms and ultimately aid in finding a cure for asthma.

In this review, we will provide the first ever summary of studies that have used complex (3D) *in vitro* co-culture models to assess lung immune cell-fibroblast interactions, and how these contribute to the various features of asthma.

## *In vitro* models used to examine fibroblast-immune crosstalk in asthma

**Figure 1:**
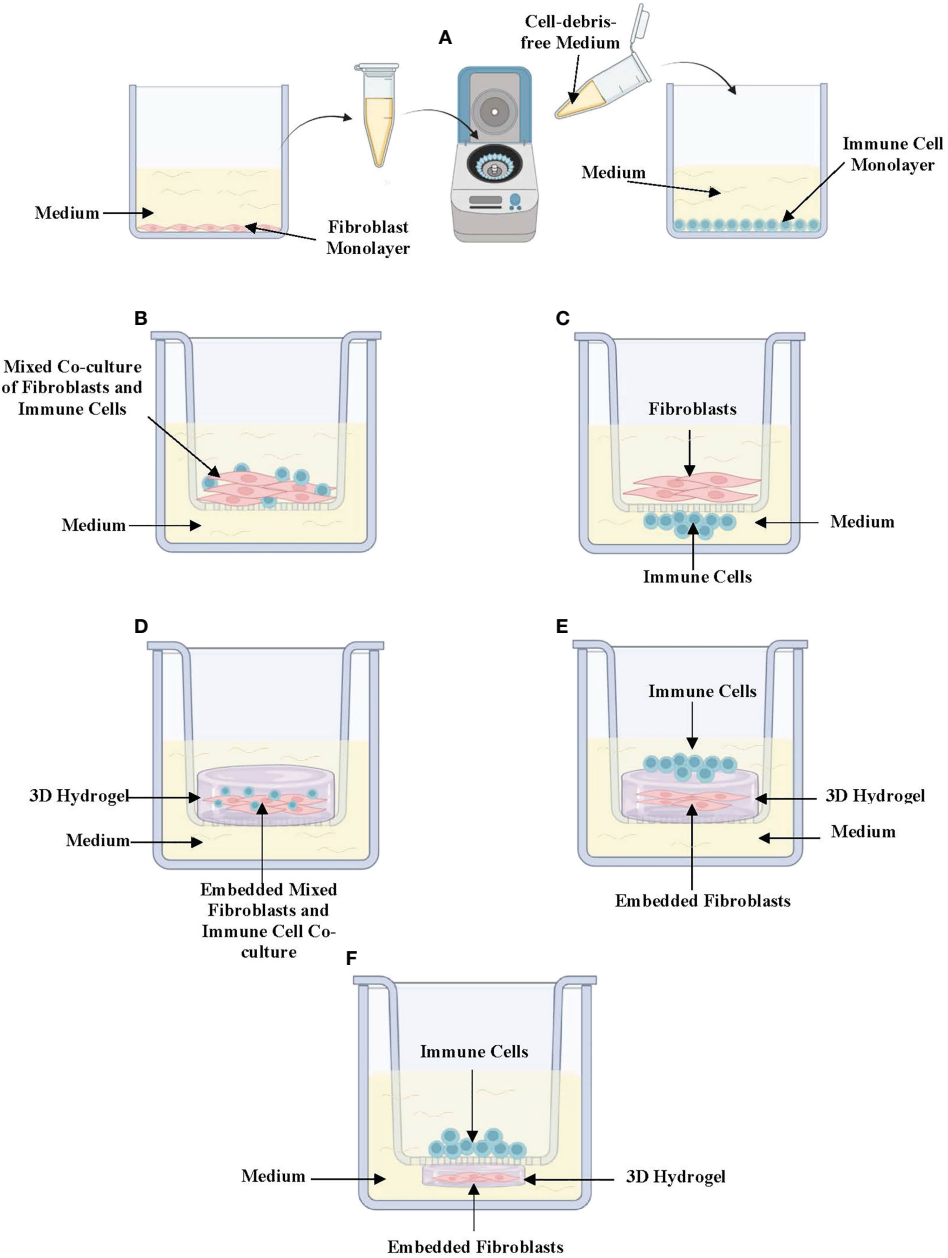
*In vitro* (3D) co-culture models of immune-fibroblast interactions in asthma. **(A)** Conditioned medium exposure treatment model in which fibroblasts and immune cells are cultured separately as 2-dimensional monolayers and cell-debris-free medium collected from one cell to be placed on the other. **(B)** Mixed co-culture model of immune cells and fibroblasts cultured in a semi-permeable transwell insert. **(C)** Direct co-culture model where fibroblasts are cultured in semipermeable transwell inserts with immune cells cultured directly underneath the insert in contact with fibroblasts. **(D)** 3D co-culture where mixed immune cells and fibroblasts embedded in a hydrogel are cultured in a semipermeable transwell insert. **(E)** 3D co-culture where fibroblasts are embedded in a hydrogel with immune cells cultured on top. **(F)** A variation of 3D co-culture where immune cells are cultured inside a semipermeable transwell insert under which a fibroblast-embedded 3D hydrogel has been cultured. Created with Biorender.com.

Different *in vitro* models have been developed in response to the demand for improved techniques that better mimic the complexity of the *in vivo* lung environment (23). Hence, there are now different types of complex *in vitro* models that are employed to specifically mimic the 3D spatial orientation of fibroblast-immune cell interactions in the *in vivo* lung-environment of asthmatics and healthy-individuals. Of these, the simplest is the conditioned medium (CM) exposure model (**Figure 1A**). In CM exposure studies, immune cells and fibroblasts are cultured separately as monolayers, and cell-debris free culture medium is harvested and incubated with the other cell-type (24). This enables the assessment of how soluble mediators released from both cell-types impact each other’s phenotype. Further, different studies have also used various variations of 2D co-cultures depending on the study objectives and the cell-cell interactions being assessed. These include co-culture models where fibroblasts and immune cells are mixed and co-cultured together inside semi-permeable transwell inserts placed in culture-wells with culture-medium at the bottom (**Figure 1B**) (24–26). Other variations involve either culturing fibroblasts inside the semi-permeable transwell inserts with immune cells cultured directly underneath the insert (**Figure 1C**) or culturing the immune cells on the culture-well surface beneath the hanging transwell insert (24, 25). In co-culture experiments, the position of fibroblasts and immune cells are interchangeable depending on which immune cell-fibroblast interaction is being studied. This is because some immune cells may be closer to the airway lumen than fibroblasts while others may be positioned beyond fibroblasts in the sub-epithelial space. 2D co-culture models are widely used due to their convenience, protocol simplicity, and ease of handling under controlled settings.

Co-culture models can be further improved to mimic more closely the 3D tissue orientation and architecture of the lung microenvironment through the addition of 3D hydrogels such as collagen-I-gels. Lung fibroblast-embedded collagen-I-gels have been adapted to study immune cell-fibroblast interactions by co-embedding immune cells and fibroblasts in collagen-I (**Figure 1D**), seeding immune cells on top of fibroblast-seeded collagen gels (**Figure 1E**) or culturing immune cells on semi-permeable transwell inserts on top of lung fibroblast-seeded 3D collagen-I-gels in culture wells (**Figure 1F**) (27). In recent years, 3D models such as lung organoid co-cultures have been established using fibroblasts and other cell types including the pulmonary epithelium and endothelium. These models have the potential to examine fibroblast-epithelial-immune cell interactions upon the addition of different immune cell types (28).

In this review, we will be summarizing the studies that have investigated fibroblast-immune cell crosstalk and how their communication contributes to airway remodeling and chronic inflammation in asthma using (3D) co-culture methods.

## Role of fibroblast-immune cell interaction in chronic inflammation in asthma

The inflammatory mechanisms that form the healthy lung’s innate immunity aids in host defence against noxious particles and pathogens in the inhaled environment of the airways (29). However, in asthma, these inflammatory mechanisms become amplified *via* aberrant responses, which partly involves abnormal interactions between immune and structural cells (e.g., fibroblasts) (29, 30). The major immune cells involved in aberrant inflammatory mechanisms in asthma include neutrophils, eosinophils, mast cells and T-cell subtypes (31–33). These immune cells play significant roles in eosinophilic/allergic asthma and non-eosinophilic/non-allergic asthma. In eosinophilic asthma there is major eosinophilia and eosinophilic degranulation which leads to the release of eosinophil cationic protein and major basic protein that leads to cytotoxicity in the airways (34, 35). Other cells involved in allergic asthma include mast cells that also undergo degranulation to release mediators such as tryptase, histamines, tumour necrosis factor (TNF)-α and various cytokines (e.g. interleukin (IL)-4, IL-5, IL-6 and stem cell factor (SCF)) (33, 36–39). In allergic asthma, T-cell differentiation into T_H_2 cells also occurs upon the presentation of antigens by dendritic cells to naïve T-cells which leads to the release of a variety of proinflammatory cytokines (IL-4, IL-9, IL-13, Granulocyte-macrophage colony-stimulating factor GM-CSF) in the airways (40). On the other hand, non-eosinophilic/non-allergic asthma involves increased neutrophilia due to the increased activity of the chemoattractant CXCL8/IL-8 and is associated with corticosteroid resistance and increased disease severity (41). Below, we summarize how these immune cells have been shown to interact directly or indirectly with fibroblasts to contribute to various aspects of inflammation in asthma.

To assess the interaction between fibroblasts and eosinophils and the role this plays in asthma pathogenesis, Esnault and colleagues exposed human lung fibroblasts (HLFs) to CM from eosinophils isolated from patients with and without asthma which had been treated with IL-5 and IL-3 to induce degranulation (42). Eosinophil-CM exposure, led to the upregulation of C3, CXCL1, IL-8/CXCL8, ICAM1 and IL-6 in HLFs, which are cytokines that cause neutrophil chemotaxis (42). Further to this, Zeng et al. established an open microfluidic model and found that normal human lung fibroblasts (NHLF) co-cultured with degranulating eosinophils had a high expression of IL-6, IL-8, granulocyte monocyte colony stimulating factor (GM-CSF) and ICAM1 due to (43). Interestingly fibroblast-derived-IL-6 and IL-8 are critical proinflammatory cytokines that activate and attract neutrophils demonstrating an importance for eosinophil-fibroblast crosstalk for neutrophil-inflammation in asthma (44–46). In line with this, an increased IL-6 concentration was found after co-culturing lung eosinophils and fibroblasts (47). This increased IL-6 was found in lung fibroblasts along with IL-11 and leukemia inhibitory factor (LIF) and shown to be due to the activity of major basic protein or proteoglycan 2, and neurotoxin derived from eosinophils (47). LIF is a pro-inflammatory cytokine in the IL-6 cytokine family. In asthma, LIF release from fibroblasts has been shown to activate eosinophils and induce degranulation (48, 49). In another study, CM from asthma-derived primary eosinophils incubated with human bronchial fibroblasts (HBF) led to an increased expression of IL-6 and IL-8 in HBFs which was found to be specifically due to the release and activity of IL-1α from eosinophils (50). Apart from the release of IL-8 and IL-6 by HBFs there was also an autocrine release of IL-8 and IL-6 by eosinophils which was dependent on eosinophilic IL-3 activity (50). IL-8 and IL-6 release in these experiments was shown to be responsible for neutrophil chemotaxis (50). Taken together, the interaction between eosinophils and fibroblasts results in an array of cytokine release (e.g., IL-6, IL-8, IL-11) that interestingly contributes to neutrophilic-inflammation in asthma.

Mast cell-fibroblast crosstalk has also been demonstrated to be crucial for asthmatic airway inflammation. In line with this, Fitzgerald et al. directly and indirectly co-cultured the human mast cell line HMC-1 with NHLFs and found a transcription factor p38 mitogen-activated protein kinases (MAPK) activation with, subsequent IL-6 production by lung-fibroblasts (51). Through comparisons between the direct and indirect co-culture-model setup, it was discovered that mast-cell-fibroblast crosstalk requires direct and physical cellular interactions for the significant increase in IL-6 by NHLFs (51). Another study by Zhao et al. using co-cultures found that chymase, an enzyme released from mast cells, caused human lung fibroblasts to release chemokines including CXCL1, CXCL5, insulin-like growth factor-binding protein (IGFBP), and CXCL6, important for neutrophilic chemotaxis (52). Therefore, in all, data from mast cell-fibroblast interaction studies corroborate that seen from eosinophil-fibroblast-crosstalk studies. This is because mast cell-fibroblast interaction studies also lead to fibroblast-derived inflammation and the production of cytokines (e.g., CXCL1, IL-6, IL-8) that drive neutrophilic chemotaxis (51, 52).

Other studies have looked at how the interaction between different T-cell subtypes and fibroblasts also contribute to inflammation in asthma. Here, Loubaki and colleagues co-cultured CD4+ T-cells with primary HBFs isolated from healthy controls and asthmatics (53). It was found that in co-culture, CD4+ T cells had an increased release of IL-17 and IL-22 and induced an increased release and mRNA expression of IL-6, IL-1β, TGF-β and IL-23 in asthma-derived HBFs compared to controls (53). This CD4+ T-cell-fibroblast interaction was further found to be due to the activity of IL-23 which stimulates HBF-IL-6 and IL-8 expression (53). This study demonstrated the importance for T-cell-fibroblast crosstalk in neutrophilic inflammation, as IL-8 and IL-6 are known neutrophil chemoattractants. In agreement with this, Loubaki and colleagues performed another study where they directly cultured asthma-derived T-lymphocytes with primary bronchial-fibroblasts (12). They found that fibroblasts activated T-cells, as shown by an increase in CDL40L expression in asthmatic T-cells (12). Further, it was also found that there was an increased IL-6 concentration in fibroblasts upon co-culture with asthma-derived T-cells compared to controls (12). Ultimately these studies show that, T-cell-lung fibroblast interactions cause release of cytokines such as IL-6, IL-8, and IL-23 which work to attract leukocytes to aid chronic inflammatory processes in asthma.

Altogether, these studies demonstrate the significance of immune-fibroblast crosstalk in asthmatic chronic airway inflammation. A surprisingly common theme between fibroblast-eosinophil, mast cell, and T-cell interactions was that these predominantly resulted in IL-8 and IL-6 inflammation which is important for neutrophil recruitment and chemotaxis. This is an important finding which seems to blur the lines between eosinophilic/allergic and neutrophilic/non-allergic asthma. This is because through crosstalk studies, it has become apparent that even during allergic asthma, interactions between cells such as eosinophils, mast cells and T-cells with fibroblasts seem to lead to neutrophilic inflammation. Such findings will not have been apparent in simple 2D-culture or animal-model studies. Since IL-6 and IL-8 (released from the resulting eosinophil, mast cells and T-cell fibroblast crosstalk) are key neutrophil chemoattractants, it would be beneficial to further assess the interactions between neutrophils and fibroblasts *in vitro*. There is currently a lack of studies assessing neutrophil-fibroblast interactions in asthma pathogenesis which may be due to inherent difficulties in the culturing and maintenance of neutrophils as these cells need to be freshly isolated from blood to be used for experiments (54). Nevertheless., neutrophils have been successfully co-cultured with airway epithelial cells to assess pathogen induced transepithelial migration in the airways (55). These methods can be potentially adapted in future studies to assess fibroblast-neutrophil crosstalk in asthma pathogenesis.

## Role of fibroblast-immune cell interaction in asthmatic airway fibrosis

The lungs of asthmatics undergo extensive remodeling that is partly characterized by airway fibrosis resulting in the thickening and narrowing of the airways (31, 56–58). Airway fibrosis involves the proliferation and migration of lung fibroblasts as well as the increased production and degradation of ECM proteins which is important for matrix turnover (6, 59, 60). Recently, fibroblasts have been shown to interact with immune cells to increase the production and degradation of ECM proteins.

Several studies have demonstrated the importance of eosinophil-fibroblast crosstalk in asthmatic airway fibrosis. Increased ECM protein synthesis and deposition by fibroblasts in the asthmatic airways is a major feature of the disease (19). In connection with this, Esnault and colleagues cultured primary HLFs in the presence of CM from asthma-derived eosinophils (42). CM from asthma-derived eosinophils upregulated *α*-smooth muscle actin (*α*-SMA), collagen I, and matrix metallopeptidases (MMPs) in HLFs. The increased production of ECM proteins was correlated with matrix turnover and degradation as well as increased ECM stiffness in the thickened asthmatic airways (42). Further, Kuwabara et al. used co-cultures to show that direct contact between eosinophils and human fetal lung fibroblasts (HFL-1) caused an increase in the fibroblast to myofibroblast differentiation, as determined by an increased expression of *α*-SMA *(* 61). They further demonstrated that TGF-β1 released from eosinophils further induced MMP-2, MMP7, MMP9 and MMP12 which are all essential for abnormal matrix turnover and myofibroblast differentiation in asthma fibrosis. In another study, Janulaityte et al. isolated asthma-derived eosinophils and added them in a suspension of culture media to the human fetal lung fibroblast (MRC-5) cell-line. In corroboration with other studies, they found that asthma-derived eosinophils caused an upregulation in the ECM proteins collagen I, collagen III, fibronectin, and elastin in MRC-5 fibroblasts (62). Asthma-derived eosinophils also stimulated an increased expression of MMP2, MMP-9, MMP-12, TGF-β1 and TGF-β2 in MRC-5 fibroblasts also in correlation with an abnormal matrix turnover and airway fibrosis (62). Furthermore, Zagai et al. isolated eosinophil cationic protein from blood donors and found that it stimulates the release of TGF-β from HFL-1 (27). TGF-β is a pleiotropic growth factor which is crucial for the differentiation of fibroblasts to profibrotic myofibroblasts which are major players in the increased production of ECM proteins during fibrosis. In summary, various studies have shown that asthma-derived eosinophils interact with fibroblasts to stimulate the over production of ECM proteins (e.g., collagen I, collagen II, fibronectin, elastin, *α*-SMA) and profibrotic cytokines such as TGF-β that are implicated in asthma fibrosis (42, 61, 62). In addition, there is significant release of MMPs involved in abnormal matrix turnover involved in the thickening and increased stiffness of asthmatic airways (61, 62).

The interaction between mast cells and fibroblasts have also been shown to result in increased ECM production. Margulis and colleagues demonstrated this by co-culturing primary mast cells or HMC-1 activated with C5a, with HFL-1 cells embedded in 3D collagen-I-gels (63). They found that fibroblasts produced a significant amount of collagen-I when cultured with primary mast cells vs HMC-1 due to the proteolytic activity of MMPs which activates TGF-β to induce matrix deposition (63). Further to this, Plante and colleagues also cultured HMC-1 cells inside the upper part of a transwell-insert and asthma-derived primary fibroblasts underneath (64). They found that mast cells produced significantly more IL-4 when cultured with asthma-derived-fibroblasts compared to controls, with a subsequent increase in fibroblast-derived procollagen proteins (64). In short, mast cell-fibroblast crosstalk results in cytokine release (e.g., IL-4, TGF-β) that ultimately upregulates collagen synthesis, an integral part of airway fibrosis in asthma.

Asthmatic airway fibrosis is largely due to increased deposition of ECM proteins as well as an imbalance between the degradation and repair of the ECM. The studies discussed in this section demonstrates how fibroblast-immune cells interactions are essential for specific features of fibrosis. Here, eosinophil-fibroblast interactions involving the release of TGF-β and MMPs cause ECM synthesis and regulate matrix turnover. Further, TGF-β release was also found to be important for mast cell-fibroblast interactions which are involved in excess ECM production in asthma (e.g., collagen-I).

## Future directions and conclusion

In this review, we provided an unbiased summary of studies that have examined the contribution of fibroblast-immune-cell crosstalk to the pathogenesis of asthma with specific emphasis on airway inflammation and fibrosis (See **Table 1**). In addition to the various *in vitro* co-culture models previously discussed, there are various applications using more advanced biological models. These include precision cut lung slices, microfluidic lung-on-a-chip systems (LOAC) and 3D lung bioprinted models, which are all being established to mimic the complex 3D *in vivo* environment of the lungs and allow for the study of more than two cell-types. Although, the addition of other cell types to these models may prevent the ability to isolate and study the specific communication between two cell types such as immune-fibroblast communication as examined in this review, triculture models such as these represent more wholistic mimics of the airway mucosa. This is because, addition of more cells, and other features of the *in vivo* environment, such as the airflow through microfluidics with these models further enable the discovery of (new) possible drug targets which hither-to were difficult to study with 2D-monolayer, animal model or (3D) co-culture systems as reviewed here.

**Table 1:** Summary of studies assessing fibroblast-immune cell interactions in asthma using co-culture models.

Fibroblast-immune cell interactions in asthmatic airway inflammation using co-culture models				
Model	Description	Mediator(s)	Finding	Ref.
Conditioned medium (CM)	Asthma & non-asthma HLFs exposed to IL-3 & IL-5-preactivated eosinophil-CM	Increased fibroblast- C3, CXCL1, IL-8, ICAM1, & IL-6	Increased cytokines are important for neutrophil chemotaxis.	(42)
Co-culture	NHLFs co-cultured with degranulating eosinophils	Increased HLF release of IL-6, IL-8, GM-CSF, & ICAM1	Inflammatory cytokines cause activation and attraction of neutrophils	(43)
Co-culture	NHLFs co-cultured with lung eosinophils	Increased fibroblastic-release of IL-6, IL-11, & LIF	MBP/PRG2 activity & eosinophil neurotoxins increase inflammatory cytokine release	(47)
Conditioned medium (CM)	CM prepared from primary asthma-derived eosinophils incubated with primary HBFs	Increased release of IL-6 and IL-8 in HBFs Increased autocrine release of IL-6 and IL-8 in eosinophils	Release of eosinophil-IL-1α increase HBF-inflammatory cytokine secretion, IL-3 activity in eosinophils regulates HBF-IL-6 and IL-8 release, IL-6 and IL-8 modulate neutrophil chemotaxis.	(50)
Direct and indirect co-culture	HMC-1 were directly and indirectly co-cultured with NHLF	Direct co-culture releases significantly more IL-6 from NHLFs than indirect	Activation of p38 MAPK regulates NHLF- IL-6 production, Direct cellular interaction required for significant NHLF-IL-6 release	(51)
Co-culture	Primary HLFs co-cultured with mast cells	Increased release of CXCL1, CXCL5, CXCL6, & IGFBP in HLFs	MC Chymase modulates HLF-chemokine release and neutrophil chemotaxis	(52)
Co-culture	CD4+ T cells co-cultured with primary HBF from asthmatic patients and healthy controls	Increased release of IL-17 & IL-22 from T-cells, Increased IL-6, IL-1β, TGF-β, and IL-23 in HBFs	IL-23 stimulates IL-6 and IL-8 release in HBFs, IL-6 and IL-8 regulate neutrophil chemotaxis, T-cell-fibroblast crosstalk modulates neutrophilic inflammation	(53)
Co-culture	T lymphocytes co-cultured with primary HBF from asthmatic patients and healthy controls	Increased expression of CDL40L in asthma-derived T-cells and IL-6 in fibroblasts after co-culture with asthma-derived T-cells	HBFs activate T lymphocytes T-cell-fibroblast crosstalk modulates neutrophilic inflammation *via* production of inflammatory cytokines	(12)
Fibroblast-immune cell interactions in asthmatic airway fibrosis using co-culture models				
Conditioned medium (CM)	Asthma & non-asthma HLFs exposed to IL-3 & IL-5-preactivated eosinophil-CM	Upregulation of *α*-SMA, collagen I, and MMPs by HLFs	Upregulation of ECM proteins increase ECM stiffness & regulates matrix turnover and degradation in asthmatic airways	(42)
Co-culture	Direct co-culture of human eosinophils and HFL-1	Increased expression of TGF-β1 by eosinophils, *α*-SMA, MMP2, MMP7, MMP9 & MMP12 by HFL-1	Eosinophilic-TGF-β1 modulates abnormal matrix turnover and myofibroblast differentiation by inducing MPP2, MMP7, MMP9, and MMP12 expression in asthma fibrosis	(61)
Conditioned medium (CM)	Asthma-derived eosinophil added in suspension to culture media that has MRC-5 lung fibroblasts	Asthma-derived eosinophils increased Collagen I, Collagen III, & fibronectin, MMP2, MMP9, MMP12, TGF-β1& TGF-β2 in MRC5s	Asthmatic eosinophils modulate abnormal matrix turnover and airway fibrosis by increasing expression of MMP2, MMP9, MMP12, TGF-β1 and TGF-β2 in MRC-5	(62)
Co-culture	Co-culture of HFL-1 and Eosinophil cationic protein	Increased release of HFL-1-derived-TGF-β1 after co-culture	TGF-β1 causes myofibroblast-differentiation that increase ECM production in asthmatic fibrosis	(27)
Co-Culture	Co-culture of HFL-1 and C5a-activated HMC-1 or primary mast cells	Increased production of collagen-I by HFL-1 co-cultured with primary mast cells	Proteolytic activity of MMPs in primary mast cells stimulate collagen-I production by HFL-1s and induces matrix deposition *via* TGF-β activation	(63)
Co-culture	Co-culture of HMC-1 and primary bronchial fibroblasts isolated from asthmatic and healthy subjects	Increased production of IL-4 by asthma-derived mast cells and procollagen proteins by fibroblasts	Mast cell-asthma-derived-fibroblast crosstalk modulates collagen synthesis and cytokine production in asthma airway fibrosis	(64)

In conclusion, the studies reviewed here demonstrate that in asthmatic airway inflammation the interaction between immune cells and fibroblasts is significantly important for the release of inflammatory cytokines such as IL-6 and IL-8 that results in neutrophil activation and chemotaxis, even when the communication is between fibroblasts and classical allergic immune effector cells such as eosinophils, mast cells and T-cells. In addition to this, we found that various studies showed eosinophil and mast cell interaction with fibroblasts through the release of TGF-β and MMPs had critical implications for increased ECM deposition and abnormal ECM turnover. These findings are critical and requires further investigation as they have interesting consequences for asthma therapeutic research. It is already known that certain asthma subtypes which are resistant to treatment with underlying neutrophilia and aberrant airway fibrosis exist (65, 66). From the studies reviewed, it could be deduced that eosinophil, mast cell and T cell- fibroblast interactions in asthmatic airways may add to the potential mechanisms in these refractory asthma subtypes. Building on such studies may provide new therapeutic targets focused on the mediators discovered through multicellular bioartificial model research (**Table 1**) and possibly provide new therapeutics for asthma.

## Author contributions

Conceptualization ETO. Original draft preparation: TF, and ETO. Further writing and edits: TF, AS, PS, AA, and ETO. Preparation of Table, Figure, manuscript citations, references, and further edits: ETO, TF, FK, DE, and BJ. All authors contributed to the article and approved the submitted version. 
